# Predictors for neonatal death in the rural areas of Shaanxi Province of Northwestern China: a cross-sectional study

**DOI:** 10.1186/s12889-015-1738-x

**Published:** 2015-04-16

**Authors:** Chao Li, Hong Yan, Lingxia Zeng, Michael J Dibley, Duolao Wang

**Affiliations:** Department of Epidemiology and Health Statistics, Xi’an Jiaotong University Health Science Center, Xi’an, PR China; School of Public Health, University of Sydney, Room 307A, Edward Ford Building (A27), University of Sydney, Sydney, NSW 2006 Australia; Department of Clinical Sciences, Liverpool School of Tropical Medicine, Pembroke Place, Liverpool, L3 5QA UK

**Keywords:** Predictors, Neonate mortality, Rural China

## Abstract

**Background:**

Almost all (99%) neonatal deaths arise in low-income and middle-income countries. Approximately 450 new-born children die every hour, which is mainly from preventable causes. There has been increased recognition of the need for these countries to implement public health interventions that specifically target neonatal deaths. The purpose of this paper is to identify the predictors of neonatal death in Type 4 rural (poorest) counties in Shaanxi Province of northwestern China.

**Methods:**

A cross-sectional study was conducted in Shaanxi Province, China. A single-stage survey design was identified to estimate standard errors. Because of concern about the complex sample design, the data were analysed using multivariate logistic regression analysis. Socioeconomic and maternal health service utilization factors were added into the model.

**Results:**

During the study period, a total of 4750 women who delivered in the past three years were randomly selected for interview in the five counties. There were 4880 live births and 54 neonatal deaths identified. In the multiple logistic regression, the odds of neonatal death was significantly higher for multiparous women (OR = 2.77; 95% CI: 1.34, 5.70) and women who did not receive antennal health care in the first trimester of pregnancy (OR = 2.49; 95% CI: 1.41, 4.40). Women who gave birth in a county-level hospital (OR = 0.18; 95% CI: 0.04, 0.86) and had junior high school or higher education level (OR = 0.20; 95% CI: 0.05, 0.84) were significantly protected from neonatal death.

**Conclusions:**

Public health interventions directed at reducing neonatal death should address the socioeconomic factors and maternal health service utilization, which significantly influence neonatal mortality in rural China. Multipara, low educational level of the women, availability of prenatal visits in the first trimester of pregnancy and hospital delivery should be considered when planning the interventions to reduce the neonatal mortality in rural areas.

## Background

The aim of the UN Millennium Development Goal 4 (MDG 4) is to reduce the mortality of children under the age of 5 by two-thirds between 1990 and 2015 [1,2]. In the past two decades, the proportion of deaths that occur in the neonatal period increased due to the important worldwide reductions in the mortality of children under the age of 5 [3]. In recent years, post-neonatal deaths have had an accelerated decrease, and deaths in the neonatal period have constituted the main component of infant mortality in many regions of the world [4]. Almost all (99%) neonatal deaths arise in low-income and middle-income countries, yet most epidemiological and other research has focused on the 1% of deaths in wealthy countries. Therefore, the inequity between wealthy and poor countries continues to increase, with lower neonatal mortality rates (NMR) and faster reductions in wealthy countries. If we neglect these challenges, 450 new-born children die every hour worldwide, which is mainly from preventable causes, and this situation is unconscionable in the 21st century. Substantial global reductions in neonatal deaths in the next decade should focus on poor countries, and they depend on increasing coverage with interventions that improve neonatal survival within the context of maternal and child health programmes [5].

China is the largest agricultural country in the world, with more than 60% of its population living in rural areas. Compared with urban populations, Chinese rural populations have a shorter life expectancy and disproportionally higher rates of overall mortality, which are associated with a broad range of health problems. These inequalities are due to the following three synergistic factors: the social determinants of health have become more inequitable, imbalances in the roles of the market and government have developed, and concerns among the public have grown about fairness in health [6,7]. In the past few decades, there has been great improvement in rural infant health; NMR among rural populations dropped from 17.3 to 10.0 per 1000 live births during period from 2004 to 2010. However, rural–urban disparities in the NMR have remained unchanged. In 2004 and 2010, the rural NMR was 2.06 and 2.44 times higher than the urban rate [8].

In low and middle income countries, a previous study revealed that the socioeconomic status (income poverty and illiteracy) and utilization of maternal health services (home delivery, no health system at grassroots level, and distant facilities) are the main intermediate determinates of neonatal mortality [9]. Therefore, understanding the specialty of intermediate determinants of neonatal mortality in Shaanxi can provide guidance for health professionals to further reduce neonatal mortality. The purpose of this paper is to identify the socioeconomic status and utilization of maternal health service predictors of neonatal death in Type 4 rural (poorest) counties in Shaanxi Province of northwestern China.

## Methods

### Study population and design

The present cross-sectional study was conducted in five Type 4 (poorest) rural counties in Shaanxi Province of northwestern China between March and April 2010. The lot quality-assurance sampling (LQAS) method, which requires a smaller sample size, was selected in this survey to determine the high NMR region and identify the predictors for neonatal death in the rural areas of Shaanxi Province.

The study sample consisted of women who delivered in the past three years (after March 2007). In the present study, we excluded multiple births and women with severe illness, such as psychiatric illness, who have been reported in several studies as being at a higher risk of neonatal death [10-12]. The five counties were located in the Guanzhong area of Shaanxi Province; the number of inhabitants and townships were similar among the five counties. The number of townships ranged from 10–20. Therefore, we used the same sampling strategy, randomly selecting 10 townships in each county. Then, 3–5 clusters that included 1–4 villages were randomly selected in each township. Each cluster includes 33 births, and the maximum sample size is 990 births in the past three years in each county. A sampling plan appropriate for classifying counties with NMR ≤9‰ (low prevalence) and ≥21‰ (high prevalence) with reasonable levels of expected error (maximum sample size of 990 and 13 allowed defects) was created with cumulative binomial probabilities. Low and high prevalence were defined according to the NMR in Type 2 (9.8‰) and Type 4 rural (21.8‰) areas of China from 2002 to 2008 [13]. The probability of finding fewer than 13 cases in a sample of 990 births from a county with NMR 9‰ is 0.932; therefore, classification of samples with 13 cases as low-prevalence populations should correctly identify approximately 93.2% of low-prevalence communities. This corresponds to an α error of 0.07. The probability of finding fewer than 13 cases in a sample of 990 births from a county with NMR ≥21‰ is 0.046; therefore, the classification of samples with >13 cases as high-prevalence populations should correctly identify nearly 96.4% of the high-prevalence counties. This corresponds to a β error of 0.046.

### Household interview and variable definition

In the present study, a face-to-face interview was conducted using a household questionnaire, which mostly included closed questions; each interview took approximately half an hour on average. The socioeconomic background, reproductive history, prenatal and postnatal health care utilization factors were collected from the interview. The number of prenatal visits was used to estimate the utilization of maternal health services. A baby who showed any sign of life was defined as a live birth. Neonatal death and early neonatal death were defined as babies who died in the first 28 days and first 7 days of life, respectively. To differentiate the differences between stillbirth (a foetus dies in the uterus after 20 weeks of pregnancy) and neonatal death, we asked women whether the baby showed any sign of life, such as voluntary movement, heartbeat, or pulsation of the umbilical cord.

We hired 25 MD students from XJTU Medical College (2 interview teams) to act as interviewers. Two experts from Sydney University trained the interviewers. During data collection, the two experts also provided supervision and technical support. This survey cooperated with the MCH (Maternal and Child Health) hospital in each county. There were two local health staff members in charge of the MCH information collection, who assisted us in contacting the township hospital and village doctors when we performed the survey in each county.

The local health staff were forewarned about the survey and asked to ensure that mothers and children would be present in their homes on the day of the survey. When the survey teams were sent to the village, they first became familiar with the terrain and distribution of the eligible households from the village doctor. They then constructed a simple map of the village and span to randomly select which direction to travel and which household to contact first. When the survey teams found an empty house, neighbours and the village doctor were asked whether a family with children aged 0–3 years or a woman delivering during the past three years lived in that house. If not, the house was registered and recorded in the logbook as having no eligible children or mothers. If children or women lived in the house, attempts were made to locate them. In most cases, the mothers and children were close to home and were called by neighbours. If mothers and children could not be located immediately, the houses were visited again at the end of the survey. To decrease the participants’ mental distress, local health staff members were asked to stay away from the place of the interview. Then, we explained the aim of the interview and reassured the participants that the conversations would be kept confidential.

The survey was approved by the Ethic Review Committee and Academic Committee, Xi’an Jiaotong University College of Medicine, Xi’an, China. Pregnant women who agreed to participate in the study signed the consent form. Relatives or neighbours signed on behalf of illiterate participants after their verbal consent.

### Statistical analysis

Summary statistics for the main outcomes were computed as the proportions or means. The NMR per 1000 live births was calculated as the number of neonatal deaths, divided by the number of live births, multiplied by 1000. A household wealth index was constructed from an inventory of 15 different household assets or facilities (bicycle, motorbike, television/VCD, type of toilet, main material used for the floors, availability of water in the house, etc.) with a principal component analysis method, and this index was categorised into thirds as an indicator for the poorest, middle income, and wealthiest households [14,15].

The effect of the sample design features on statistical inference, such as stratification, clustering, and weighting, should be seriously considered. However, most analysts routinely ignore these issues, resulting in biased estimates of the standard errors as well as an increased possibility of Type I errors. Therefore, a single-stage survey design was specified for the data analysis. The variables of the cluster and county were identified, respectively. The predictors of neonatal death were screened through univariate logistic regression. Crude odds ratios (ORs) and their 95% CIs were obtained. The analysis of the feeding covariate was restricted to singleton births who survived the first 24 h (the possibility that the breastfeeding pattern was affected by serious illnesses that lead to death). Considering there were 13 neonatal deaths within 24 h that would not be included in the analyses, the breastfeeding covariate was not added in the multivariable regression. Multivariable analyses to identify the predictors of neonatal deaths were developed according to the model from Mosley and Chen [16]. The model identified a set of proximate determinants, including socioeconomic determinates and specific diseases that directly influence the risk of NMR. Because of the association between the utilization of maternal health services and specific disease, we added the variables of the socioeconomic and utilization of maternal health services into the logistic model. Only the covariates that were associated with the outcome (P < 0.2), after potential confounders were considered, were retained. Finally, the variables of the socioeconomic determinates (maternal age, highest level of maternal education, household wealth index, and whether it was the first child) and utilization of maternal health services (distance to the delivery location, delivery location, and whether the mother visited the ANC during the first trimester) were entered into the logistic regression model as a series of dummy variables. There were no other confounders entered into the logistic regression model. In each case, the referent category is identified in the table. The 95% confidence intervals for risk of neonatal death were calculated using the binomial distribution. We used Stata version 12.0 (StataCorp LP, College Station, Texas 77845, USA) for all analyses.

## Results

During the study period, a total of 4750 women who delivered in the past three years were randomly selected for interview in the five counties. Overall, 4800 single live births (94 women delivered 2 babies in the past three years) and 54 neonatal deaths were identified. Twenty-seven households refused to participate, and 74 households could not be contacted.

### Maternal baseline characteristics and health service utilization

The mean age of the women was 27.9 years. The reproductive history of the pregnant women reflected China’s “one child policy”, and 2578 (54.1%) women were having their first delivery. Lin You County was the poorest and had the lowest educational level among the five counties; 66.4% women lived in the poorest households, and 3.4% women were illiterate. Bing County was the secondary poorest and most illiterate, with rates of 39.5% and 1.2%, respectively (Table 1).

**Table 1:** **Baseline characteristics of pregnant women by county**

	**County**					**Total sample**
	**Xun Yi**	**Bing**	**Lin You**	**Qian Yang**	**Zhou Zhi**	
Number of pregnant women	949	919	956	937	945	4706
Maternal age mean(SD)*	28.2(5.2)	28.1(5.5)	28.0(4.6)	27.5(5.0)	27.7(4.9)	27.9(5.1)
<20	9(0.9)	15(1.6)	9(1.0)	13(1.4)	12(1.3)	58(1.3)
20-35	832(87.8)	799(87.1)	882(92.9)	809(89.7)	857(91.1)	4179(89.7)
>35	107(11.3)	104(11.3)	95(6.1)	80(8.9)	72(7.6)	421(9.0)
Women’s education						
None	5(0.6)	10(1.2)	30(3.4)	5(0.6)	3(0.3)	53(1.2)
Primary school	193(21.8)	249(30.6)	382(43.8)	197(22.0)	121(13.1)	1142(26.0)
Junior high school or higher	688(77.6)	555(68.2)	461(52.8)	694(77.4)	801(86.6)	3199(72.8)
First pregnancy						
Yes	545(57.4)	405(44.1)	491(51.4)	568(60.7)	541(57.3)	2548(54.1)
Household Wealth Index						
Poorest	301(31.7)	363(39.5)	635(66.4)	162(17.3)	115(12.2)	1576(33.5)
Middle income	381(40.2)	298(32.4)	247(25.9)	333(35.5)	305(32.3)	1564(33.2)
Richest	267(28.1)	258(28.1)	74(7.7)	442(47.2)	525(55.5)	1566(33.3)

Comparison of baseline characteristics of the women delivered after March 2007 enrolled in survey trial by counties, Shaanxi Province, China 2010–04. Frequency and percentage in bracket are reported.

Values are number (%) of women (singleton babies only) unless otherwise mentioned.

*Reported as mean and SD (standard deviation).

The mean of prenatal visits during pregnancy was 4.67. The utilization of prenatal health services was best in Qian Yang. The means of the prenatal visits in first, second and last trimesters of pregnancy were 1.23, 2.20 and 3.22, respectively. Conversely, the number of prenatal visits was lowest in Bing, and the means of prenatal visits in first, second and last trimesters of pregnancy were 0.77, 1.05 and 1.61, respectively. Most women delivered their new-borns in the county-level hospital; 3881 (83.2%) women delivered in a county-level hospital, and only 141 (3%) women delivered at home (Table 2).

**Table 2:** **Timing and frequency of prenatal visit, delivery care and time to initiation of breastfeeding by county**

	**County**					**Total sample**
	**Xun Yi**	**Bing**	**Lin You**	**Qian Yang**	**Zhou Zhi**	
Number of pregnant women	949	919	956	937	945	4706
Number of prenatal visits during pregnancy, mean(SD)*	3.52(1.68)	3.43(1.67)	4.90(2.32)	6.58(3.04)	5.07(2.84)	4.67(2.63)
Number of prenatal visits in 0-3month of pregnancy	0.73(0.63)	0.77(0.67)	1.02(0.65)	1.23(0.76)	0.81(0.73)	0.91(0.71)
Number of prenatal visits in 4-6month of pregnancy	1.14(0.74)	1.05(0.78)	1.64(0.98)	2.20(1.15)	1.69(1.14)	1.54(1.05)
Number of prenatal visits after 7 month of pregnancy	1.66(1.01)	1.61(0.95)	2.24(1.34)	3.22(1.96)	2.56(1.82)	2.24(1.58)
Delivery place						
Home	56(6.1)	38(4.2)	11(1.2)	4(0.4)	32(3.4)	141(3.0)
Township	155(16.7)	134(14.6)	30(3.2)	5(0.5)	75(8.0)	399(8.6)
County	648(69.9)	696(76.1)	882(92.7)	870(93.3)	785(83.6)	3881(83.2)
Above county	68(7.3)	47(5.1)	28(2.9)	54(5.8)	47(5.0)	244(5.2)
Time to initiation of breastfeeding						
No feeding	129(13.9)	166(18.7)	43(4.7)	87(10.8)	158(17.4)	583(13.2)
≤1 h	5(0.5)	4(0.5)	7(0.8)	36(4.5)	17(1.9)	69(1.6)
1-24 h	48(5.2)	69(7.8)	184(20.2)	222(27.6)	154(17.0)	677(15.3)
>24 h	744(80.4)	646(73.0)	678(74.3)	459(57.1)	577(63.7)	3104(70.0)

*Reported as mean and SD (standard deviation).

### Neonatal deaths

**Figure 1:**
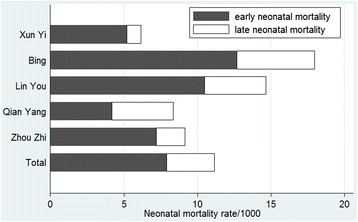
Permillage of neonatal mortality rate, early neonatal mortality and late neonatal mortality among five counties.

A total of 54 out of 4800 new-borns (96 women delivered more than one baby in the past three years) had died by Day 28. Thirty-eight of these deaths occurred during the first week of life. The point estimation of NMR in Shaanxi Province is 11.3%; the early NMR is 7.9%, accounting for 69.9% of neonatal deaths. Bing and Lin You counties had the highest NMR among the five counties. The numbers of neonatal deaths in Bing and Lin You counties were 17 and 14, and more than 13 allowed defects (Figure 1).

### Predictors of neonatal death

In the univariate analyses, the following five factors had statistical significance: multiparity, educational level, prenatal visit in the first trimester of pregnancy, delivery place and time to the initiation of breastfeeding. Having an educational level of junior high school or higher had reduced the odds of neonatal death with an OR value equal to 0.15 (95% CI: 0.04-0.57). There was a 2.5-fold (OR = 2.49; 95% CI: 1.48-4.19) increase in the odds of neonatal deaths for women who did not have a prenatal visit during the first trimester of pregnancy compared to those who did. The odds of neonatal death were 3-fold higher in neonates born to multiparous women (OR = 3.43; 95% CI: 1.84-6.40) compared to neonates born to nulliparous women. In addition, the time to the initiation of breastfeeding within 24 hours (OR = 0.04; 95% CI: 0.01-0.34) or after 24 hours (OR = 0.36; 95% CI: 0.20-0.65) was associated with reduced odds of neonatal death compared to the no breastfeeding group. Giving birth in the county-level hospital also significantly reduced the odds of neonatal death with an OR value equal to 0.30 (95% CI: 0.12-0.75), and the ORs of the township and above county-level hospital were 0.35 (P = 0.083) and 0.22 (P = 0.089), respectively.

In multivariable regression, after controlling for socioeconomic factors (maternal age, household wealth index and distance to the health facility), excluding for breastfeeding (this was not added in the multivariable regression), multiparity, educational level, prenatal visit in the first trimester of pregnancy, and delivery place remained significantly associated with neonatal death. The adjusted OR of the maternal age and household wealth index were different from the unadjusted OR, but the associations were not significant. The variables of multiparity (95% CI: 1.34-5.70), junior high school or higher educational level (95% CI: 0.05-0.84), having at least one prenatal visit in the first trimester of pregnancy (95% CI: 1.40-4.40), and giving birth in the county-level hospital (95% CI: 0.04-0.86), were associated with lower odds of neonatal death (Table 3).

**Table 3:** **Maternal and newborns baseline associated with odds of neonatal death** ^**a**^

**Variables**	**Number of live births n = 4800**	**Number of deaths (NMR) n = 54**	**Unadjusted OR (95% CI)**	**Adjusted OR (95% CI)**
***Socioeconomic background***				
Maternal age				
≤35	4189(91.0)	48(1.15)	1.0	1.0
>35	415(9.0)	6(1.45)	1.26(0.56-2.84)	0.53(0.18-1.56)
First pregnancy				
Yes	2536(54.5)	14(0.55)	1.0	1.0
No	2116(45.5)	40(1.89)	3.43(1.84-6.40)	2.77(1.34-5.70)
Women’s education				
None	52(1.2)	3(5.77)	1.0	1.0
Primary school	1122(25.8)	20(1.78)	0.31(0.08-1.16)	0.28(0.07-1.19)
Junior high school or higher	3171(73.0)	28(0.88)	0.15(0.04-0.57)	0.20(0.05-0.84)
Household Wealth Index				
Poorest	1553(33.4)	23(1.48)	1.0	1.0
Middle income	1551(33.3)	13(0.84)	0.57(0.28-1.16)	0.76(0.38-1.52)
Richest	1548(33.3)	18(1.16)	0.79(0.44-1.41)	1.19(0.64-2.19)
***Maternal health care utilization***				
Women receiving any prenatal visit in the first trimester of pregnancy				
Yes	3000(69.8)	26(0.87)	1.0	1.0
No	1297(30.2)	28(2.16)	2.49(1.48-4.19)	2.49(1.41-4.40)
Delivery place				
Home	136(3.0)	5(3.68)	1.0	1.0
Township	394(8.5)	5(1.27)	0.35(0.10-1.15)	0.23(0.05-1.03)
County	3839(83.3)	42(1.09)	0.30(0.12-0.75)	0.18(0.04-0.86)
Above county	242(5.2)	2(0.83)	0.22(0.04-1.26)	0.17(0.02-1.36)
Distance to the delivery place				
≤5 km	1058(23.3)	9(0.85)	1.0	1.0
>5 km	3487(76.7)	45(1.29)	1.52(0.75-3.07)	3.61(0.93-14.04)
Time to initiation of breastfeeding^b^				
No feeding	565(12.9)	13(2.30)	1.0	
>24 h	3069(70.1)	27(0.88)	0.36(0.20-0.65)	
≤24 h	745(17.0)	1(0.13)	0.04(0.01-0.34)	

*Abbreviations*: ANC, antenatal care; NMR, neonatal mortality rate.

Values are number (percentage) unless otherwise mentioned.

Logistic regressions were adjusted for clustering difference among counties.

^a^There are no more other confounders included in the multivariable logistical regression model.

^b^Restricted to singleton births who survived the first 24 h after March 2007(N = 4693) and not added into multivariable logistical regression.

## Discussion

The historically used LQAS design is still an appealing technique for the rapid evaluation of preventive programs, especially in the rural areas of developing countries [17]. Our study is a rarity in Shaanxi Province as a community-based, LQAS method that measured the burden of neonatal mortality in a rural area. The participants were a random sample of pregnant women from five counties in the study area. The fieldwork lasted almost 2 months to capture any variation in the maternal and new-born characteristics. The collaboration with well-trained interviewers from XJTU Medical College, who had professional medical knowledge and interviewing skills, should have resulted in high-quality data from a representative sample of eligible mothers.

The average NMR in our study was 11.3%, which is a little lower than that reported for the neighbouring Gansu Province (17.8%) [6]. Consistent with other studies, most neonatal deaths occurred in the first week of life because neonates are at the highest risk of infection in this period [18,19]. The NMR is different among the five counties; Bing County and Lin You County were determined as high-NMR counties. The high-NMR in two counties may be due to multiple reasons, such as hospitals still undergoing construction, the lack of prenatal diagnosis and emergency treatment, and the delivery technique of the hospital staff. These two counties are also the poorest and have the lowest education levels among women. In addition, Bing and Lin You counties were also the farthest away from the nearest city, which may reduce the access to maternal health services and thus cause different NMR among the five counties. Most neonatal deaths (17) were in Bing County. One explanation is that many coal mines were developing when the field work was implemented there; therefore, pollution of the air and river might be the main causes of the high NMR. Unpublished results from a large trial in Bing County that we previously conducted revealed a high prevalence of birth defects (25.6%) in Bing County, which could provide indirect evidence for the causes of neonatal deaths observed in this study.

### Predictors of neonatal death

A previous study revealed that the socioeconomic factors (income poverty and illiteracy) and utilization of maternal health services (home delivery, no health system at grassroots level, and distant facilities) are the main intermediate determinates of neonatal mortality in low- and middle-income counties [9]. In the present study, we found that multiparous mothers, home delivery, low education levels of women and not receiving any prenatal visit in the first trimester of pregnancy were associated with increased odds of neonatal death.

In detail, we found that multiparous women are at a higher risk of neonatal death, and a similar result was also found in the United States. The elevated risk of neonatal death in multiparous women may be due to more chronic disease can contribute to higher rates of neonatal mortality in multiparas [20]. In addition, multiparous women had a higher risk of inadequate use of prenatal care or shorter interpregnancy intervals, which can result in an increased risk of adverse perinatal outcomes were another possible explanations [21]. The low educational levels of women could result in a handicap for receiving health information as well as in recognizing bad signs of pregnancy and delivery [22,23].

Other studies have reported that the odds of neonatal mortality were higher in the group of mothers with inadequate prenatal care, illustrating the importance of prenatal care during pregnancy [4,24]. In the present study, we also found an association between prenatal health care utilization and neonatal death. Our results suggest that prenatal health care utilization in the first 12 weeks of pregnancy is important for reducing NMR. We found evidence that prenatal health care in the first trimester significantly reduces NMR. To the best of our knowledge, similar results have not been described elsewhere. We also analysed prenatal health care in the second and last trimesters of pregnancy in our univariable and multivariable analyses, but there were no statistically significant effects (data not shown). It could be that starting early predicts a greater emphasis on pregnancy visits antenatally. The results also suggested that the prenatal health care in the first trimester could identify the risk factors of neonatal death and provide suggestions for pregnant women to correct their behaviors during pregnancy as soon as possible.

We found some weak evidence that giving birth in a county-level hospital was associated with decreased odds of neonatal death compared to those who gave birth at home. This was because home delivery with an unskilled attendant may increase the risk of neonatal death through increasing the risk of birth asphyxia and the risk of sepsis during the neonatal period. Another study reported that most county-level or higher-level hospitals can deliver the elements of skilled birth attendance and emergency obstetric care, which are essential for ensuring neonatal survival, such as foetal monitoring, caesarean section, hygienic delivery care, neonatal resuscitation, and parenteral antibiotics; conversely, many township hospitals do not match to these criteria [9]. Additionally, previous studies reported that planned home births were associated with significantly elevated neonatal mortality rates, and greater proportion of deaths attributed to respiratory distress and failed resuscitation. Home births experienced significantly more 5-minute Apgar scores < 7 as compared to low-risk term hospital births [25].

In addition, the results of the univariable analyses indicate that early breast milk (within 24 h) is significantly associated with reduced neonatal deaths. However, there were insufficient numbers of deaths to determine effects on the initiation time of breastfeeding within 1 hour. We did not find any neonatal death in the group of initiation time within 1 hour compared to when we combined the groups of within 1 hour and 24 hours. However, this could also provide indirect evidence that the initiation time of breastfeeding within 1 hour could be a preventive factor for neonatal death. A larger study also found an increased risk of neonatal death with delayed initiation of breastfeeding. They reported that the initiation time of breastfeeding was associated with neonatal infection [26].

In general, all of the independent predictors of neonatal mortality identified in the present study have been previously documented. Our findings suggest that public health interventions directed at reducing neonatal death could address the socioeconomic factors and maternal health service utilization. Previous studies have reported many factors that are significantly associated with neonatal death, but we failed to find them in the present study. For example, a cohort study conducted in a rural area in Burkina Faso found an association between a shorter distance to the nearest health facility and increased odds of neonatal death, which was mainly because of poor local traffic conditions and vehicles, influencing the access to health services [27]. However, a similar traffic situation was not apparent in the present five counties. Meanwhile, the township hospitals have been established to administer primary care since the economic reforms, and the poor accessibility of maternal health care utilization was not the major problem in the rural areas of Shaanxi Province. In addition, another study found that lack of prenatal care was associated with increased risks for the individual antenatal high-risk conditions. Prenatal care had a beneficial effect on maternal anemia and intrapartum fever [28]. We did not find that the number of prenatal visits was significantly associated with neonatal death in the present study (data not shown), showing that the quality of prenatal visits was low in rural China.

We should highlight several potential limitations of the present study. First, the cross-sectional study design could not be used to determine causation. In addition, the potential reporting bias inherent with self-reported information could not be avoided in the present study. Second, we did not include all possible confounders (such as maternal nutrition status) in our analyses. In this paper, we only focus on the factors of socio-economic and maternal health service utilization. Third, these findings may not be generalizable to populations with different socioeconomic characteristics.

Our current analysis shows that specific and important determinants of neonatal mortality lie at community, health system and social level. A multi-pronged strategy of health system strengthening, behaviour change is required to address neonatal issues. The health system strengthening should not be restricted to county-level facilities but has to include in township-level facilities in rural western China.

## Conclusion

The distribution of NMR among the five poor counties is not balanced. The burden of neonatal mortality is high in some rural area of Shaanxi Province. Our findings call for increased health facility access and improved quality of care in childbirth services in rural areas of Shaanxi Province. Women with a low educational level who have previously gone into labour may require greater attention during the pregnancy and longer time in labour. A prenatal visit in the first pregnancy and early breastfeeding should be promoted. Further studies are needed to gain further insight about the neonatal deaths in Shaanxi Province.
